# A Systematic Review and Meta-Analysis: Do We Still Need Microscope Surgery in Hepatic Artery Anastomosis to Decrease the Incidence of Complications in Living Donor Liver Transplantation?

**DOI:** 10.7759/cureus.48112

**Published:** 2023-11-01

**Authors:** Beshoy Elkomos, Philopateer Alkomos, Rao Junaid Saleem, Guirgis Ebeidallah, Joseph Hanna, Amr Abdelaal

**Affiliations:** 1 General Surgery, Ain Shams University, Cairo, EGY; 2 Medicine, Ain Shams University, Cairo, EGY; 3 General and Emergency Surgery, Northwick Park Hospital, London, GBR; 4 Acute and Emergency Medicine, Royal Derby Hospital, University Hospitals of Derby and Burton NHS Foundation Trust, Derby, GBR; 5 General Surgery, Manchester Foundation Trust, Manchester, GBR

**Keywords:** end-to-end vascular anastomosis, liver transplantation, microsurgery, hepatic artery, living donor liver transplant (ldlt)

## Abstract

Hepatic artery thrombosis (HAT) is the most serious vascular complication after liver transplantation (LT). Moreover, in comparison to deceased donor liver transplantation (DDLT), hepatic artery (HA) anastomosis is more challenging in living donor liver transplantation (LDLT) with a lot of controversial topics about the use of microscopic surgery. We aimed to compare the use of microscopic and loupe surgery in HA anastomosis in adult and pediatric LDLT to decrease the incidence of vascular complications. We searched PubMed, Scopes, Web of Science, and Cochrane Library for eligible studies from inception to April 2023 and a systematic review and a meta-analysis were done. According to our eligibility criteria, 10 studies with a total of 1939 patients were included. In comparison to microscopic surgery, loupe anastomosis has a similar incidence of HAT (thrombosis, risk ratio (RR) = 0.96, 95% CI = 0.26-3.48, P = 0.95). In addition to that, no significant difference was detected between the two types in terms of stenosis, decreased blood flow and hospital stay (decreased blood flow, RR = 0.68, 95% CI = 0.01-86.65, P = 0.88), (stenosis, RR = 1.81, 95% CI = 0.19-17.21, P = 0.60), (hospital stay, mean deviation (MD) = 1.16, 95% CI = -3.79-6.11, P = 0.65). However, the anastomotic time was longer in the case of microscopic surgery (anastomotic time, MD = 24.09, 95% CI = 7.79-40.39, P = 0.004). With an equal incidence of complications and longer anastomotic time, there is no added benefit of the routine use of microscopic surgery in HA anastomosis in LDLT.

## Introduction and background

With an incidence of 7% in deceased donor liver transplantation (DDLT) and up to 13% in living donor liver transplantation (LDLT), hepatic artery (HA) complications after liver transplantation are considered a major problem [1]. These complications can lead to catastrophic repercussions including liver necrosis, abscess, biliary stricture and liver cell failure [2]. Moreover, HA thrombosis (HAT) is associated with 50% mortality and up to 70% incidence of retransplantation [3].

In addition to that, to tackle the deceased organ scarcity, decrease the waiting list for transplantation and possibly better survival outcomes, the need for LDLT has increased worldwide [4,5]. Due to smaller and shorter vessels in comparison to DDLT, hepatic anastomosis in LDLT is considered a technically challenging step [6].

Some studies adopted the idea of using microscopic hepatic artery anastomosis to decrease the incidence of complications [7,8]. In addition to that, a recent single-arm review suggests that microscopic anastomosis reduces vascular complications and improves outcomes after liver transplantation [9]. However, other recent studies suggest that HA anastomosis using loupe achieves similar outcomes with less operative time [10,11].

The aim of this study is to compare the outcomes of microscopic versus loupe HA anastomosis in LDLT.

## Review

Patients and methods

Search Strategy

The databases (PUBMED, Web of Science, Scopus and Cochrane Library) were searched from inception to April 2023 using a combination of these words: liver transplantation, living donor, and hepatic artery. Two authors (Elkomos B and Alkomos P) reviewed all articles independently. we started by reviewing the article based on the abstract followed by reading the full manuscript according to our inclusion and exclusion criteria. In addition to that, an additional search was done using Google Scholar to detect any additional literature that could be included in our analysis.

Inclusion and Exclusion Criteria

To begin with our inclusion criteria, the included studies should be: 1. retrospective, prospective or randomized controlled trials (RCTs); 2. articles designed to compare the outcomes of microscopic versus loupe hepatic artery anastomosis; 3. anastomosis only in living donor liver transplantation either adult or pediatric; 4. articles written in English. However, these studies were excluded: 1. case reports, case series or reviews; 2. studies lacking a comparative group; 3. anastomosis done in deceased donor liver transplantation.

Outcomes of Interest

Our primary outcome was to compare the incidence of complications (hepatic artery thrombosis, stricture, and decreased blood flow) for those who underwent microscopic and loupe hepatic artery anastomosis. Our secondary outcome was to compare the two groups regarding the anastomotic time and hospital stay.

Data Extraction

For each of the included studies, the following data were extracted: data related to study design (author, year of publication, country of transplant, sample size, suture material, technique and the methods used for anastomosis either microscopic or loupe), patient details (age, sex and Model for End-Stage Liver Disease (MELD) score), operative details (operative time, and anastomotic time), hepatic artery complications (thrombosis, decrease blood flow and stricture). Two authors extracted the data (Elkomos B and Alkomos P).

Statistical Analysis

We followed the Cochrane Handbook for Systematic Reviews of Interventions [12], which is recommended by the Cochrane Collaboration. The pooled risk ratio (RR) and their 95% confidence interval (CI) were calculated for each hepatic artery complication, and the mean difference was calculated for operative time and hospital stay using a fixed effect model. However, if there was a significant heterogeneity (I2 > 40%), the random effect model was used. The results are considered statistically significant if the p < 0.05. All the calculations of this meta-analysis were done using Review Manager 5.4 (Cochrane Collaboration, Oxford, UK).

Results

Characteristics and Quality Assessment of Eligible Studies

**Figure 1:**
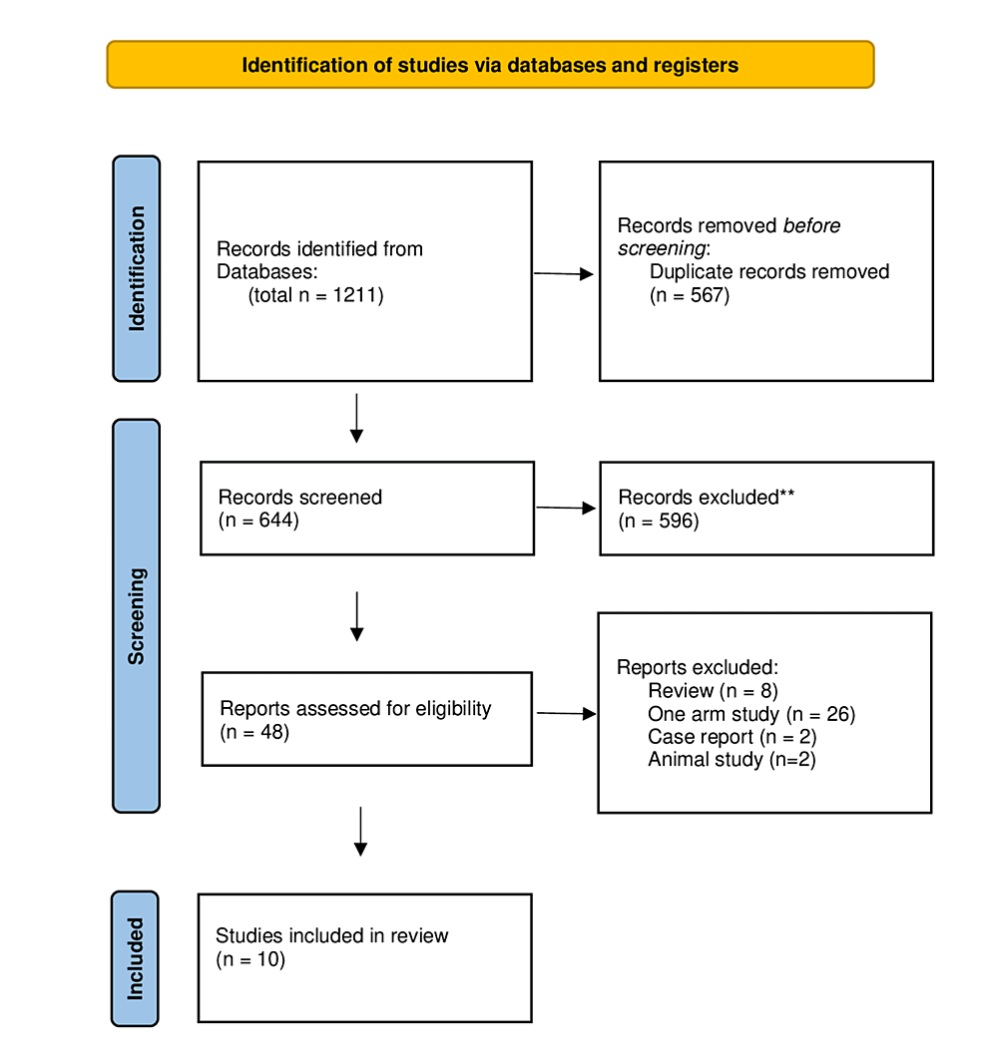
Preferred Reporting Items for Systematic Reviews and Meta-Analyses (PRISMA) flow diagram

As shown in the Preferred Reporting Items for Systematic Reviews and Meta-Analyses (PRISMA) flow diagram (Figure 1), 1211 articles resulted from searching the database using this search string: liver transplantation, living donors, pediatric and hepatic artery. After careful selection according to our eligibility criteria, 10 studies [8,11,13-20] with a total of 1939 patients who underwent living donor liver transplantation using either microscope or loupe hepatic artery anastomosis were included in our study (710 patients with microscopic surgery and 1229 patients loupe) (Table 1).

**Table 1:** Basic data of the included studies. (*) The results are presented as means and standard deviation. (**) The results are presented as median and range MELD: Model for End-Stage Liver Disease, HA: hepatic artery

Studies: author, year, country	Study design	Study period	Technique	Arm	Magnification power	Suture material	Sample size (n)	Age (yr)	Gender: Male/ Female(n)	MELD score	HA size
Tanaka et al (1993) Japan [13]	Retrospective study	1990-1992	N/A	Microscopic	10—20 ×	8-0 9-0 Prolene	26	N/A	N/A	N/A	N/A
			N/A	Loupe	3 ×	7-0 polybutester	7	N/A	N/A	N/A	N/A
Furuta et al (1997) Japan [14]	Retrospective study	1990-1995	interrupted	Microscopic	N/A	8-0 or 9-0 nylon	40	N/A	N/A	N/A	N/A
			interrupted	Loupe	N/A	8-0 or 9-0 nylon	10	N/A	N/A	N/A	N/A
Amin et al (2009) Egypt [15]	Retrospective study	2001-2007	N/A	Microscopic	N/A	8–0 nylon	102	N/A	N/A	N/A	N/A
			N/A	Loupe	N/A	N/A	30	N/A	N/A	N/A	N/A
Liang et al (2012) China [16]	N/A	2006-2010	interrupted	Microscopic	6-10 ×	8–0 nylon	67	N/A	N/A	N/A	N/A
			N/A	Loupe	3.5 ×	8–0 nylon	6	N/A	V	N/A	N/A
Yagi et al (2012) Japan [17]	Retrospective study	1996-2008	interrupted	Microscopic	N/A	7-0 or 8-0 Prolene	94	N/A	N/A	N/A	N/A
			continuous	Loupe	3.5 ×	7-0 or 8-0 Prolene	115	N/A	N/A	N/A	N/A
Li et al (2017) Taiwan [18]	Retrospective study	2002-2016	interrupted	Microscopic	12 ×	8-0 Prolene sutures	25	N/A	N/A	N/A	N/A
			continuous	Loupe	4.5 ×	7-0 Prolene	741	54 (21-75) **	N/A	1769 (5-47) **	N/A
Jwa et al (2018) Korea [19]	Retrospective study	2012-2016	interrupted	Microscopic	N/A	8-0 Nylon	136	50.2±9.5 *	90/46	17.6±10.8 *	2.07±0.11 *
			interrupted	Loupe	N/A	8-0 Nylon	101	53.7±8.1 *	73/28	16.3±8.9 *	2.06±0.10 *
Tan et al (2020) Singapore [8]	Retrospective study	2006-2018	interrupted/2 layers	Microscopic	10 ×	8-0 & 9-0 Ethilon	20	N/A	N/A	N/A	N/A
			N/A	Loupe	N/A	N/A	19	N/A	N/A	N/A	N/A
Chang et al (2021) South Korea [11]	Retrospective study	2014-2020	interrupted	Microscopic	N/A	9-0 nylon	150	52.6±9.5 *	98/52	16.7±10.2 *	N/A
			continuous	Loupe	5 ×	8-0 polypropylene	150	55.3±10.1 *	114/36	16.2±9.5 *	N/A
Huang et al (2023) USA [20]	Retrospective study	2012-2020	interrupted	Microscopic	6-10 ×	8-0 Nylon	50	57.5±14.5 *	22/28	N/A	N/A
			N/A	Loupe	N/A	N/A	50	58 ±11.8 *	29/21	N/A	N/A

Primary Outcomes

**Figure 2:**
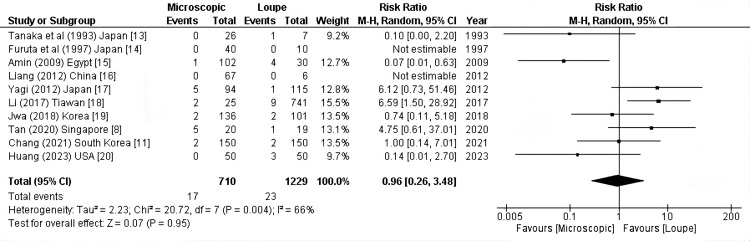
Hepatic artery thrombosis

HA thrombosis: According to the pooled results of 10 studies (1939 patients), no significant difference in the incidence of hepatic artery thrombosis between microscopic and loupe surgery (RR: 0.95, 95% CI = 0.26-3.48, P = 0.95; I2 =66%) (Figure 2).

**Figure 3:**
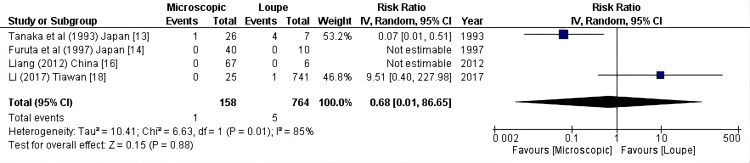
Decreased blood flow

Decreased blood flow: Regarding the decrease in blood flow after anastomosis, as reported by four articles (922 patients), the pooled results showed no difference between the two modalities in terms of decreased blood flow (RR: 0.68, 95% CI = 0.01-86.65, P = 0.88; I2 = 85%) (Figure 3).

**Figure 4:**
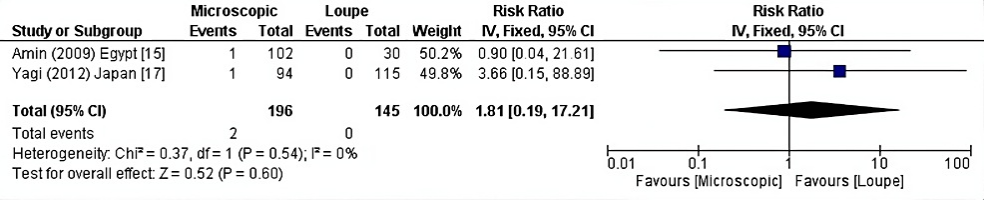
Stricture

Stricture: In addition to that, the incidence of stricture was equal in the two modalities as per two of the included studies (341 patients) (RR: 1.81, 95% CI = 0.19-17.21, P = 0.60; I2 = 0%) (Figure 4).

Secondary Outcome

**Figure 5:**
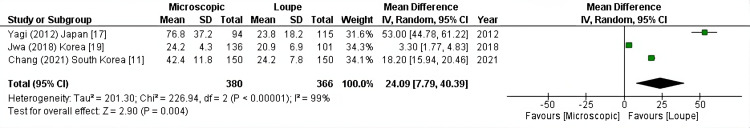
Anastomotic time

Anastomotic time: According to three of the included studies (746 patients), the anastomotic time was longer in case of microscopic surgery (mean difference 24.09, 95% CI = 7.79-40.39, P = 0.004; I2 = 99%) (Figure 5).

**Figure 6:**
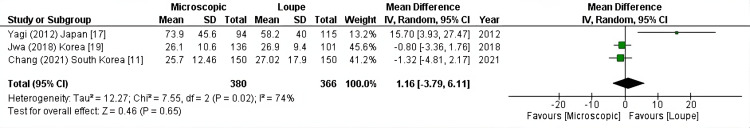
Hospital stay

Hospital stay: However, no difference could be detected between the two modalities in terms of hospital stay (mean difference 1.16, 95% CI = -3.76-6.11, P = 0.65; I2 = 74%) (Figure 6).

Discussion

Our study showed no significant difference between microscopic and loupe hepatic artery anastomosis in terms of the incidence of thrombosis, decreased blood flow, stricture and hospital stay. However, microscopic HA anastomosis is associated with longer anastomotic and operative time compared to loupe anastomosis. 

One key factor for surgeons to operate accurately is a clear visualisation of the anatomical structures. In 1921 the Swedish scientist Carl Nylen designed the first monocular surgical microscope to perform ear surgery. Two years later his colleague Gunnar Holmgren developed the binocular microscope [21]. From this time, the use of microscopic surgery has increased in different fields of surgery including vascular, plastic, neuro and transplant surgery. The advantages of microscopic surgery in vascular anastomosis include high magnification power of up to 40 times, gives the opportunity to do anastomosis in delicate vessels less than 3 mm using sutures as thin as 9/0 and 10/0 that in terns believed to decrease the incidence of complications [22]. However, this type of anastomosis will need longer operation time, more experienced surgeons and more place in the operative field in comparison to conventional anastomosis. On the other hand, loupe vascular anastomosis has been adapted as a conventional way of vascular anastomosis that gives less magnification power nevertheless it provides safe, feasible and easier methods for vascular anastomosis.

In liver transplantation, vascular anastomosis is more challenging because the vessels are deeply seated; more than 15 cm from the abdominal wall with a short stump and continuous movement because of the respiration and heartbeats. In addition to that, in comparison to DDLT, blood vessels in LDLT are smaller, thinner and could be multiple which makes the anastomosis harder. In 1992, according to 14 cases of HA anastomosis in LDLT performed by Keiichiro et al., microscopic surgery had a lower incidence of complication in comparison to loupe anastomosis [23]. In addition to that, in 2004, by using a surgical microscope with a magnification of 12× to 16× for hepatic artery anastomosis in 14 cases of segmental paediatric liver transplantations, Guarrera et al. reported no cases of HAT happened [24]. Moreover, according to Amin et al. [15], despite having its own challenges, microscopic HA anastomosis decreased the incidence of HAT from 13% in case of loupe anastomosis to 0%. This has been explained by the magnification power of the microscopic surgery and the presence of a plastic surgeon who is only responsible for performing HA anastomosis. 

On the other hand, in 2017, according to a study of 741 cases of LDLT, Li et al. stated that surgical loupe leads to the speedy anastomosis with a low incidence of HAT [18]. Moreover, one year later, a Korean study reported no significant difference in the incidence of complications between the two modalities in addition to that surgical loupe can offer simple vascular anastomosis even with less experienced surgeons [19]. In addition to that, in pediatric LDLT, with an average hepatic artery diameter of 2.38 mm, Yagi et al. reported that non-microscopic anastomosis has a lower incidence of overall complications in comparison to microscopic anastomosis (2.6% vs. 11.7%, P < 0.005) [17]. The pooled results of the included studies showed similar incidences of HAT, decreased blood flow after anastomosis and HA stricture for both modalities.

Marubashi et al. reported better results of HA anastomosis using a loupe with the advantage of decreasing the operative time [25]. In addition to that, Yagi et al. reported a 100-minute decrease in the operative time associated with the use of surgical loupes [17]. The pooled results of the included studies showed longer anastomotic associated with microscopic surgery.

To our knowledge, it is the first meta-analysis to compare the outcomes for microscopic and loupe hepatic artery anastomosis in LDLT. In addition, all the studies in the database comparing the two modalities were included. However, we must admit the presence of some limitations in our study; the suturing technique (continuous or interrupted) was not compared in this study. Moreover, no RCT was found to be included in our study. In addition to that, there was a high heterogeneity among primary outcome parameters.

## Conclusions

With an equal incidence of complications and longer anastomotic time, there is no added benefit of the routine use of microscopic surgery in hepatic artery anastomosis in LDLT (moderate GRADE of evidence). Loupe anastomosis offers a great alternative to microscopic surgery in HA anastomosis.
